# Microstructure and composition evolution of He charged solid-gas nanocomposite films of different matrix elements during thermal annealing in vacuum

**DOI:** 10.1038/s41598-025-06889-8

**Published:** 2025-07-02

**Authors:** Asunción Fernández, M. Carmen Jiménez de Haro, Dirk Hufschmidt, Olga Montes, Thierry Sauvage, F. Javier Ferrer, Amaël Caillard, Pascal Brault, Anne-Lise Thomann

**Affiliations:** 1 Institute of Materials Science of Seville (CSIC-Univ. Seville), Avda. Américo Vespucio 49, 41092 Sevilla, Spain; 2 CEMHTI Laboratory, CNRS-UPR3079, 1D Avenue de La Recherche Scientific, 45071 Orléans, France; 3 National Center of Accelerators, CNA (Univ. Seville, J. Andalucía, CSIC), Avda. Tomas Alva Edison 7, 41092 Seville, Spain; 4 CNRS, GREMI-UMR7344, 14 Rue d’Issoudun, 45067 Orléans, France

**Keywords:** Helium assisted magnetron sputtering of Co, Si and Zr, ^4^He and ^3^He charged thin films, Helium release by thermal annealing, Nanobubbles and nanopores, Microstructural characterization, IBA analysis, Materials science, Nanoscience and technology

## Abstract

Sputtering of cobalt, silicon and zirconium in a helium magnetron discharge (MS) is reported as a bottom-up procedure to obtain He-charged films (i.e. ^4^He and ^3^He filled nanopores encapsulated in the matrix material). Composition and microstructural analyses are presented from ion beam analysis (IBA) and scanning and transmission electron microscopies (SEM and TEM). Helium desorption was investigated by IBA in a dedicated chamber for “in situ” thermal evolution in vacuum. The simultaneous recording of the helium and matrix-element signals shows different behaviors of the different matrix elements (i.e. Co, Si and Zr) and deposition conditions (i.e., DC or RF discharge modes and dynamic or quasistatic vacuum). Effusion, blistering, delamination and flaking have been observed for the different samples leading to the formation of nano-porous/nanostructured thin films. The methodology is being envisaged as a process for nanostructured thin-films fabrication with potential applications.

## Introduction

The predominant characteristic of noble gas atoms implanted in most solids through ion beam irradiation, across a wide energy spectrum (500–100 eV), is their high heat of solution. This results in nearly zero solubility and the precipitation of gas atoms, forming small ‘bubbles’^1–5^. Helium, in particular, has been extensively studied due to its technological importance in examining damage in nuclear reactor materials^6,7^. Helium plasma irradiation of metal surfaces was reported to produce fuzzy nanostructures together with He bubbles formation^8–10^. Also the growth process of aluminum thin films by MS, using different Ar/He plasma mixtures, showed that in the He rich plasma regime the film nanostructure can be controlled from He bubbles formation (closed pores) to a highly porous fiber-form nanostructur^11^. At the ICMS laboratory in Seville special attention was paid to the investigation of helium incorporation and He filled pores formation during magnetron sputtering (MS) deposition of thin films of silicon^12,13^ and cobalt^14^. Also, other authors have reported He incorporation in Ti^15,16^ and C^17^ during MS deposition using He as process gas. The development of what we named “solid–gas” nanocomposite films allowed to propose these films as solid-targets of noble gases for nuclear reactions^18–21^ and spectroscopic studies^22^. This avoids the use of cryogenic or high-pressure cells facilitating usage, reducing energy straggling effects and simplifying geometry for calculations. In addition, due to their intrinsic nanoporosity (filled with gas), the silicon films fabricated by MS in pure He show a reduced refractive index^12^ and have been used for the fabrication of optical devices^23^. In another previous work we have demonstrated improved activity for the catalytic combustion of hydrogen in Pt-Cu porous films prepared by MS deposition in helium followed by dealloying. The effect has been attributed to enhanced lattice strain effects associated to the nanoporosity of the as prepared films^24^.

Building on the above-described state of the art, we present in this article the study of Helium release by heating in vacuum the He-charged films fabricated by magnetron sputtering in helium plasma. Different matrix elements and different deposition conditions were investigated. In two previous works first results were presented for the case of Si:He films^25,26^. The simultaneous recording by IBA analysis of the helium and the matrix-element signals during “in situ” annealing experiments are presented here for the first time. Also, the microstructure of the films before and after annealing was analyzed by electron microscopy (SEM and TEM). The article aims to presents the different mechanism of He release for three investigated matrix elements (Co, Si and Zr) evolving from He filled nanocomposite to different nanoporous structures. The work also aims to show the fabrication of porous structures out of the different He-charged films. This contributing to the actual growing knowledge on the fabrication and applications of films and coatings obtained by magnetron sputtering using Helium as process gas.

## Results

### Solid–gas nanocomposite films: preparation and characterization (by IBA)

Table 1 summarizes the nomenclature of the investigated samples along with their MS deposition parameters: substrate onto which the film was growth, deposition time, gas pressure, discharge power and evaluated deposition rates. Table 2 shows the absolute determination of He and “matrix element (M)” area densities, given in 10^15^ atom/cm^2^ (TFU units), for the total film thickness of the as prepared samples. Atomic ratios of He to matrix elements have been also included in the table. The solid–gas nanocomposite nature of the “as prepared” films is therefore demonstrated by the IBA analysis results.

**Table 1:** Nomenclature and deposition parameters for investigated samples (“as deposited”).

Sample nr Description	Substrate	Deposition time (h)	Sputtering gas and pressure (Pa)	Power (dc or rf) (W)	Deposition rate^a)^ (nm/min)
E49 **1.** Co:He-RF	Silicon	4	4 (He)	200 (rf)	4.9 ± 0.1
E101 **2.** Si:He-DC^b)^	Glassy carbon Silicon	4	4.8 (He)	100 (dc)	8.0 ± 0.1
E102 **3.** Si:He-RF^b)^	Glassy carbon Silicon	4	4.8 (He)	150 (rf)	5.7 ± 0.2
E69 **4.** Si:^3^He-RF(static)^b), c)^	Glassy carbon Silicon	4	5 (^3^He)	150 (rf)	4.8 ± 0.1
G1 **5.** Zr:He + Ar-DC	Silicon	1	1 (95% He and 5% Ar	300 (dc)	16 ± 0.3
G2 **6.** Zr:He-DC	Silicon	2	1 (He)	410 (dc)	2 ± 0.3

^a^Calculated from deposition time and the thickness determined by SEM. Note that the target to substrate distance during deposition was 8 cm for Co, 10 cm for Si and 12 cm for Zr.

^b^Samples grown on: (i) glassy carbon for IBA analyses and (ii) silicon to facilitate TEM lamella preparations.

^c^This sample was prepared using a low gas consumption procedure in static operation mode. See^19^.

and section “Methods” for details.

**Table 2:** Areal density for Helium and matrix elements (Co, Si, Zr) and average porosity for the total thickness of investigated films.

Sample nr Description	Thickness^a)^ (µm)	Areal density (10^15^ at/cm^2^)			Atomic ratio ^4^He/M	Atomic ratio ^3^He/M	Porosity (%)
		^4^He	^3^He	M: Co/Si/Zr			
E49 **1.** Co:He-RF	1.19 ± 0.02	925 ± 50	–	5150 ± 100	0.18	–	47 ± 1
E101 **2.** Si:He-DC	1.92 ± 0.03	3250 ± 100	–	5000 ± 100	0.65	–	46 ± 2
E102 **3.** Si:He-RF	1.37 ± 0.05	2350 ± 50	–	3250 ± 75	0.72	–	50 ± 3
E69 **4.** Si:^3^He-RF(static)^b)^	1.16 ± 0.01	–	917 ± 7	4378 ± 48	–	0.20	22 ± 0.5
G1 **5.** Zr:He + Ar-DC	0.96 ± 0.02	890 ± 45	–	2980 ± 60	0.30	–	27 ± 1
G2 **6.** Zr:He-DC	0.25 ± 0.02	125 ± 6	–	586 ± 12	0.21	–	45 ± 4

^a^Measured from SEM cross-section (at different positions and cleaved areas).

^b^The “static” methodology aims to reduce the ^3^He gas consumption^19^.

Considering the matrix element total areal densities in at/cm^2^ and the film thicknesses, the density of the films, in number of atoms per cm^3^, were evaluated. These values were compared to reported densities for amorphous-Si, Co and Zr. The reduction in density is therefore used to quantify the % of porosity and the results are included in Table 2. Film thicknesses have been determined from “cross-sectional” SEM images showed in the following section.

In our previous work for Si films in^25^, we found that oxygen incorporation during film growth promotes He diffusivity and therefore reduces He accumulation. In this study we worked under optimum conditions to minimize possible impurities bellow 5 at% for these films. For the case of Zr the oxygen incorporation is relevant and needs to be considered more in detail. It can be estimated from RBS results and it is of the order of 15 at% in sample 5 and 45 at% in sample 6, which is the most porous. Actually, the incorporation of oxygen can take place during the growth, and is promoted when the deposition rate is low, which is the case in 100% He (sample 6). However, it may also occur after the growth due to air exposure when porosity is high and included closed nanopores but also open porosity. In this last case, the role of oxygen incorporation on the growth process itself would be less relevant for sample 6. For additional information we refer to Ref. 11 were the growth of Aluminum films, in pure He and different He + Ar mixtures were investigated. In this article it was also shown that the plasma drastically changed, turning from 95% He to 100% He compositions^11^.

### *Thermal annealing of solid–gas nanocomposite films: “*In situ*” IBA analyses coupled to microstructural characterization before and after annealing*

For each of five investigated samples the IBA signals of helium and the corresponding matrix element were simultaneously recorded during annealing in the dedicated DIADHEM chamber. In order to facilitate the visualization and quantification of the matrix element signals, Co and Zr films were grown on silicon wafer substrates while glassy carbon substrates were used for the Si films. SEM (top and cross views) and TEM images were also recorded for the samples before and after annealing.

**Fig. 1:**
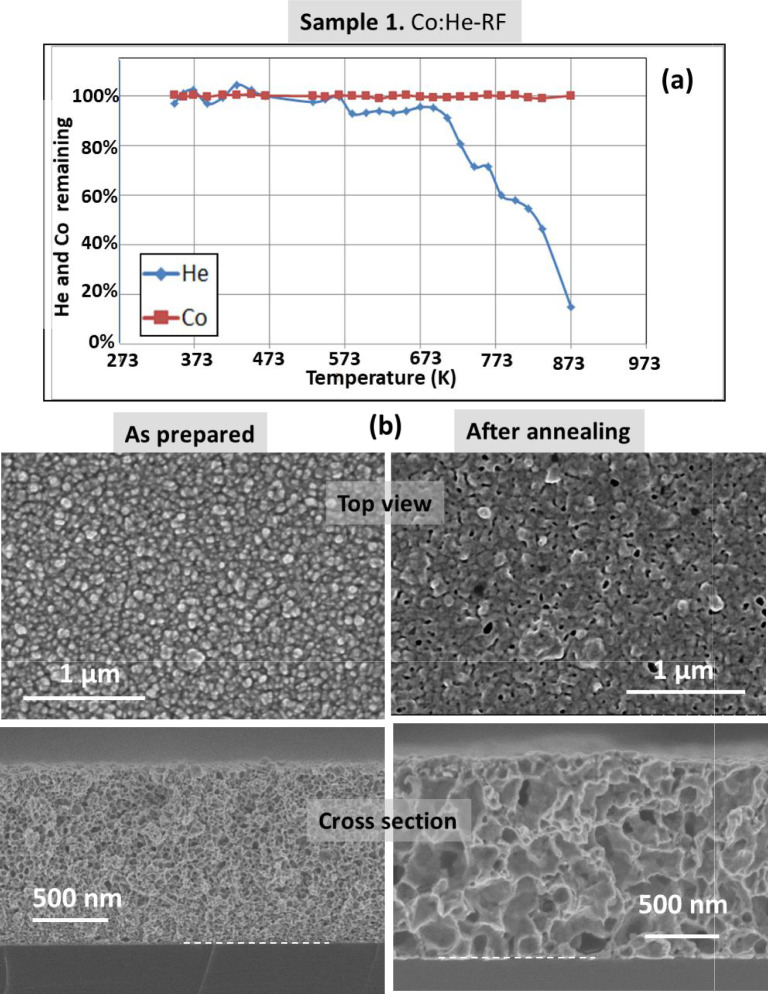
(**a**) Helium and Cobalt content evolution for sample **1** derived from proton-EBS spectra during *in-situ* annealing in vacuum at indicated temperatures. (**b**) SEM top-view and cross-section images of sample **1** as prepared and after annealing in vacuum.

**Fig. 2:**
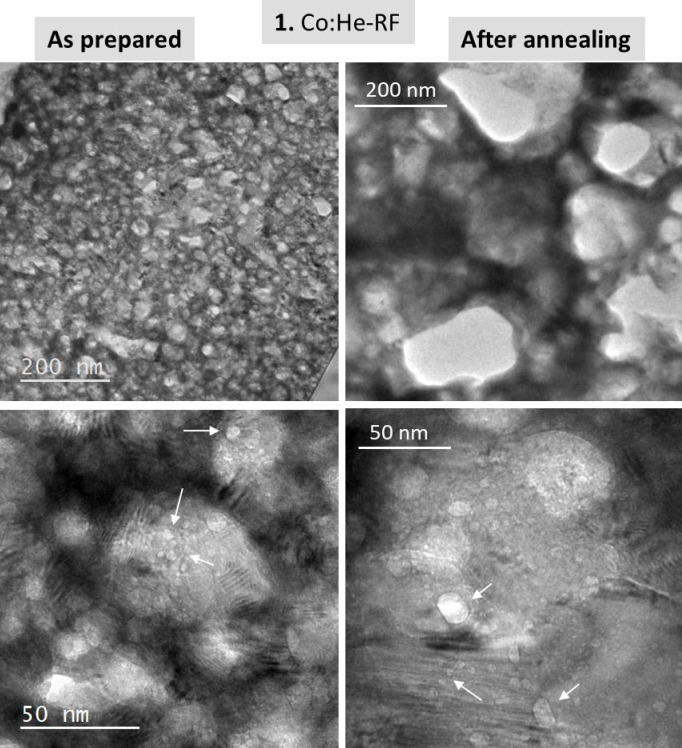
TEM cross-section images of sample **1** as prepared and after *in-situ* annealing.

Figure 1 shows the results for the cobalt film (sample 1). Even at the lower resolution achieved with the SEM microscope, top and cross images of the as prepared film show strong porosity and nano-structuration. Thermal annealing in vacuum produces the He release without modification of the cobalt IBA signal (Fig. 1a). A noticeable He release starts at 673 K. The nanoporous structure is maintained, both in the “top” and “cross-sectional” images although an increase in pores size was clearly observed. These results can be described as a helium effusion mechanism associated to a matrix modification due to ductility of the cobalt nanoporous structure under He release pressure at the reached temperatures of up-to 873 K^27^. Complementary results (before and after annealing) are presented in Fig. 2 from TEM images of the Co films at low (top) and high (bottom) magnifications. The strong porosity and the increase in pore sizes after annealing is also clearly visualized at the higher resolution achievable by TEM. In addition, the higher magnification TEM images allow to visualize the characteristic contrast associated to nanobubbles/(He-filled nano-pores)^13^. Arrows are used in the high magnification images to indicate some of these nano-pores. Some larger ones appeared in the annealed sample and are clearly faceted. This effect is associated to the crystalline character of the Co film (see Fig. 1sa in the supporting information file). Smaller and round nanobubbles are also clearly observed both in the “as prepared” and “annealed” samples.

**Fig. 3:**
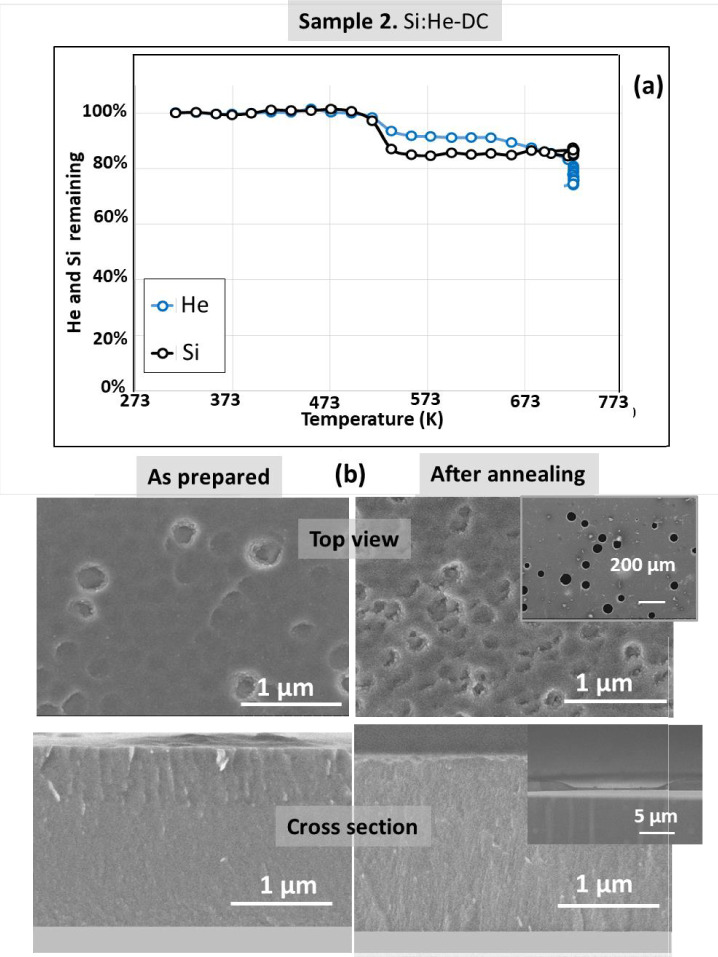
(**a**) Helium and Silicon content evolution for sample **2** derived from proton-EBS spectra during *in-situ* annealing in vacuum at indicated temperatures. (**b**) SEM top-view and cross-section images of sample **2** as prepared and after annealing in vacuum.

**Fig. 4:**
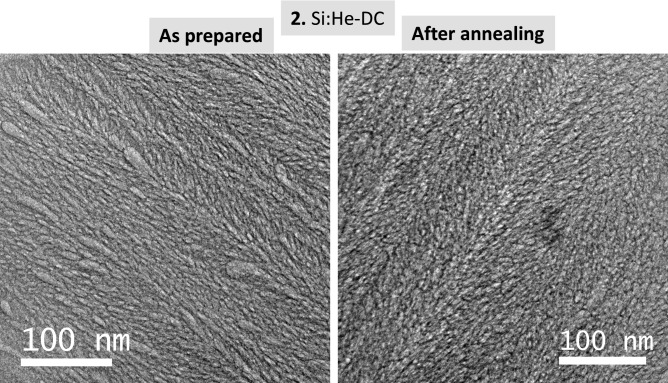
TEM cross-section images of sample **2** as prepared and after *in-situ* annealing.

Figure 3 shows the results for the Si film grown in DC mode (sample 2). In comparison to cobalt, at the resolution achieved by SEM, the “cross-sectional” images for this sample does not show pores. For the visualization of nanopores TEM resolution is needed due to the smaller pore sizes in this sample. First relevant observation in Fig. 3 is a moderate blistering in the top view low magnification SEM image for the as prepared film (sample 2) that become stronger after annealing. Also after annealing both smaller and bigger blisters are observed. An image of one blister can be seen in the high magnification inset in the SEM “cross-sectional” image after annealing. For sample 2 blistering produces the local detachment of the Si film coupled to He release. This leads to the simultaneous decrease of the Si and Helium IBA signals observed in Fig. 3a under annealing from ca. 515 to 573 K. For further heating helium release occurs continuously without modification of the silicon signal. Blistering appears to be a consequence of the higher stress reported for the Si films fabricated in DC conditions^25^. Figure 2s in supporting information shows two enlarged “cross-sectional” SEM images of representative areas showing different sizes of blisters for sample 2 after annealing. Complementary results (before and after annealing) are presented in Fig. 4 from TEM images of sample 2. The characteristic structure associated to numerous nanobubbles/nano-pores^12,13^ is clearly observed. The pores for the Si matrix are not faceted due to the amorphous character of the Si films. See Fig. 1sb in supporting information showing the absence of characteristic diffraction peaks for Si. In spite of the blistering effect, the pore size and shape distribution are similar in the remaining Si film before and after He release.

**Fig. 5:**
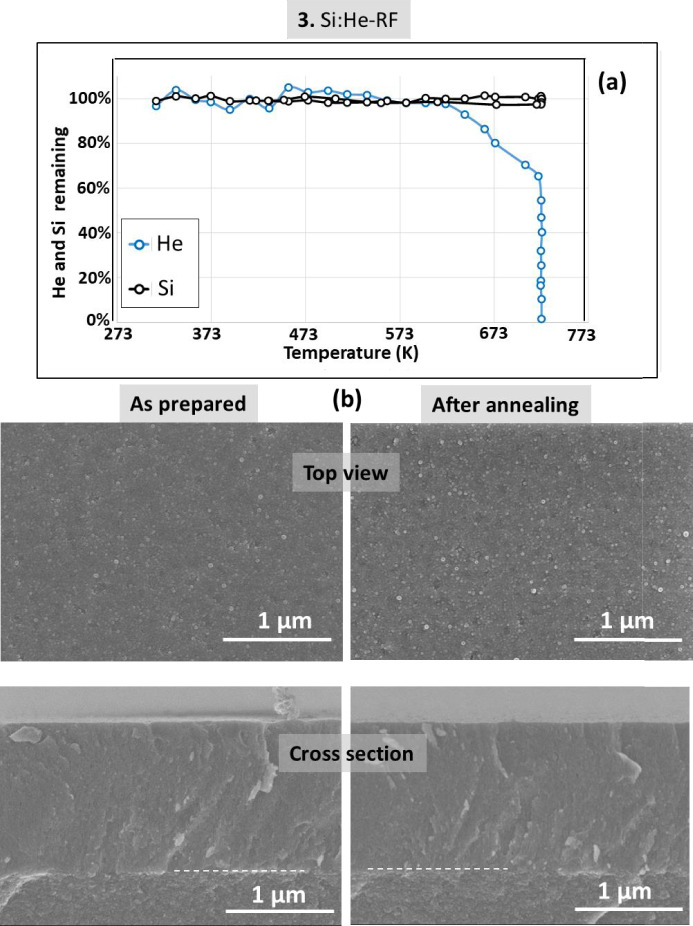
(**a**) Helium and Silicon content evolution for sample **3** derived from proton-EBS spectra during *in-situ* annealing in vacuum at indicated temperatures. (**b**) SEM top-view and cross-section images of sample **3** as prepared and after annealing in vacuum.

**Fig. 6:**
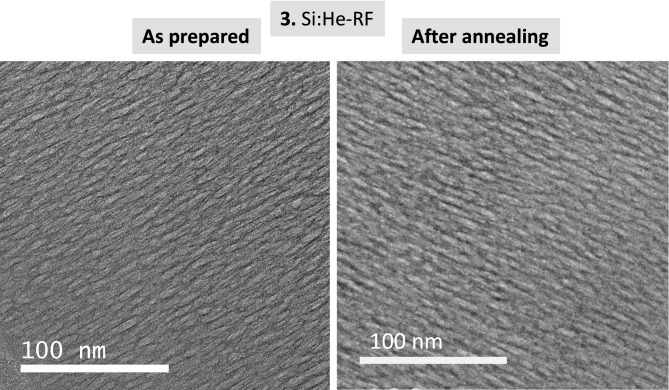
TEM cross-section images of sample **3** as prepared and after *in-situ* annealing.

Figure 5 shows the results for the Si film grown in RF mode (sample 3). As compared to the sample fabricated in DC mode, the absence of blistering in this sample is obvious from the top view images after annealing. This correlates with the observation, during the thermal annealing in vacuum, of He release (starting at 625 K) without modification of the silicon IBA signal (Fig. 5a). An effusion mechanism is therefore proposed without producing significant changes in the nano-porous structure of the Si matrix. For the visualization of nanopores, TEM resolution is needed. Figure 6 shows representative TEM images of sample 3 showing the characteristic structure associated to nanobubbles/nano-pores. Similar pore size and shape distributions are observed before and after He release. The pores are not faceted in agreement with the amorphous character of the Si films.

**Fig. 7:**
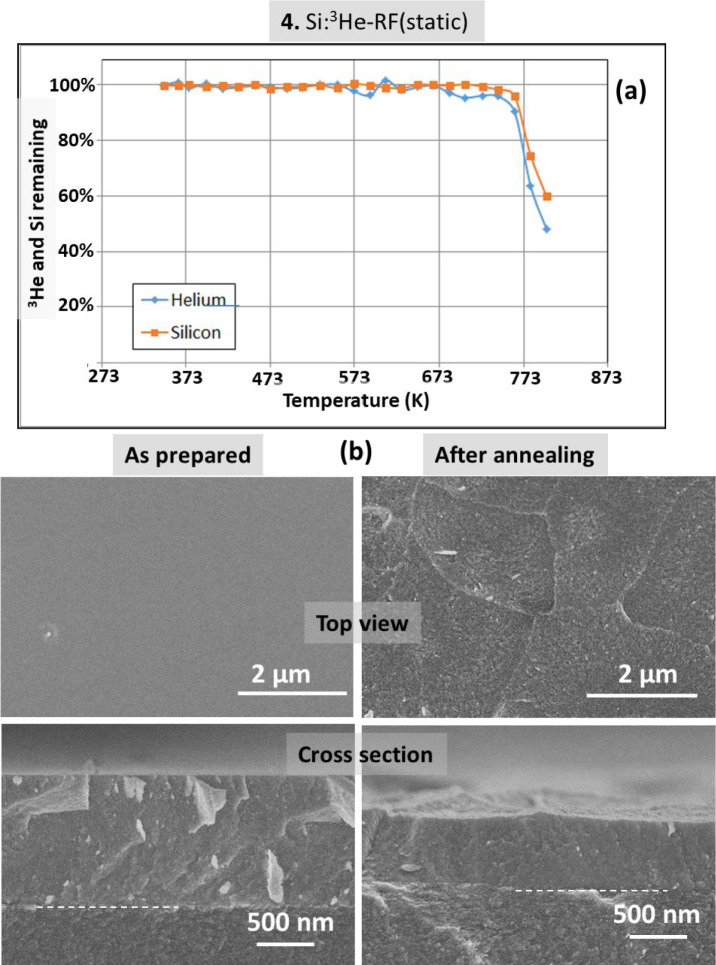
(**a**) Helium 3 and Silicon content evolution for sample **4** derived from proton-EBS spectra during *in-situ* annealing in vacuum at indicated temperatures. (**b**) SEM top-view and cross-section images of sample **4** as prepared and after annealing in vacuum.

**Fig. 8:**
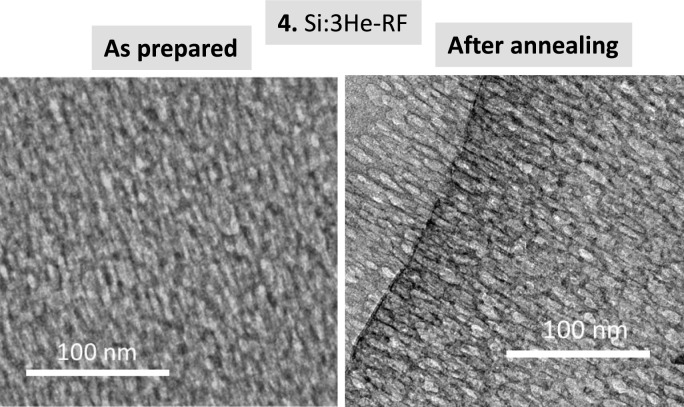
TEM cross-section images of sample **4** as prepared and after *in-situ* annealing.

Figure 7 shows the results for the Si film grown using the ^3^He isotope as the process gas (sample 4). Note that in the rest of the samples ^4^He was always used. The “top-view” and “cross-sectional” SEM images clearly show a delamination effect during the thermal annealing in vacuum. This effect also explains the observed simultaneous decrease of the He and Si signals during the in-situ IBA analysis (Fig. 7a). The behavior is attributed to the particular case of the use of ^3^He as process gas. During fabrication of ^4^He charged films by MS deposition, we work in a dynamic operation mode^12,14^. In this mode the He pressure in the chamber is stablished by introducing a given gas flow and controlling the aperture of a throttle valve that is connected to a continuous pumping system^12,14^. For the case of the ^3^He isotope a quasi-static method is used to strongly reduce the consumption of the costly ^3^He^19,20^. In this method the throttle bulb is almost closed and the helium working pressure is stablished with a needle valve employing a very low gas flux. We tentatively attribute this delamination effect to a different temperature regulation during film growth. Thermal conductivity of Helium is high and the minimum He flux used in quasi-static mode leads to a higher temperature at the substrate (486 K for sample 4 as compared to 393 K for sample 3). The resulting delamination effect in this work is however relevant in the context of the fabrication of exfoliated silicon films^28,29^. Complementary results (before and after annealing) are presented in Fig. 8 from TEM images of sample 4. The characteristic structure associated to numerous nanobubbles/nano-pores is observed. The pores are again not faceted due to the amorphous character of the Si films. A delamination border is also shown for the annealed sample in the higher resolution TEM image.

**Fig. 9:**
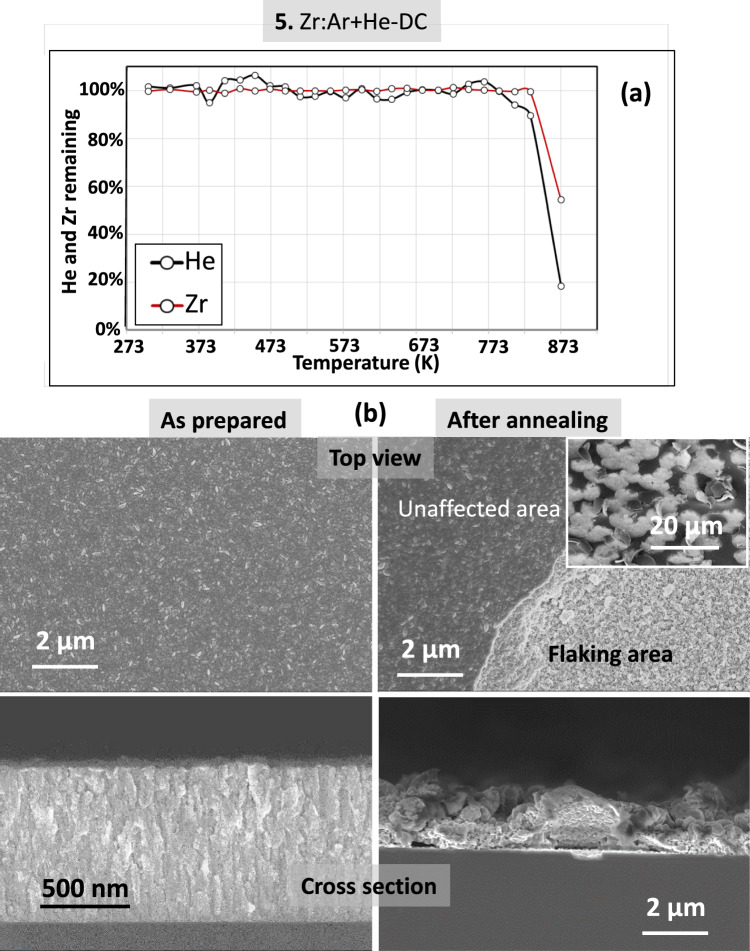
(**a**) Helium and Zirconium content evolution for sample **5** derived from proton-EBS spectra during *in-situ* annealing in vacuum at indicated temperatures. (**b**) SEM top-view and cross-section images of sample **5** as prepared and after annealing in vacuum.

**Fig. 10:**
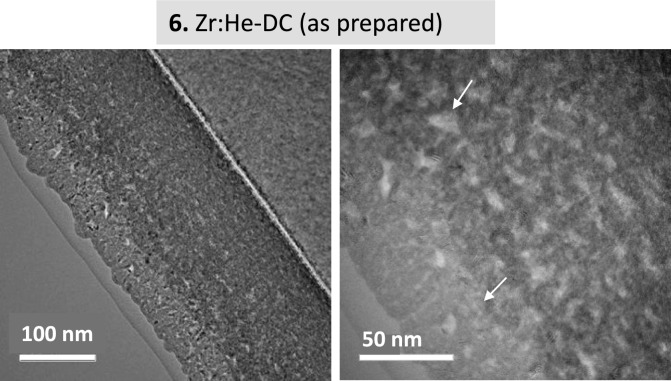
TEM cross-section images of sample **6** as prepared.

Figure 9 shows the results for the Zr film grown in 95%He + 5%Ar mixture and in DC mode (sample 5). The “top-view” and “cross-sectional” SEM images evidence a columnar “as-prepared” film. After annealing, a notorious flaking effect is evidenced. Large (several to 10 µm) almost spherical interconnected zones are visible. They exhibit a rough surface state, whereas unaffected surface state is found in areas where the film is still present. The extended damages caused to the film by the release of both He and part of the Zr are clearly observed on the “cross-sectional” SEM images. This flaking effect correlates with the simultaneous sharp decrease of the Zr and He signals as measured by *in-situ* IBA analyses (Fig. 9a). The Zr films are stable up to temperatures as high as 773 K when the helium release starts accompanied by flaking of the film. Due to the micrometer scale of the blisters forms by annealing on Zr films, TEM images are only provided in Fig. 10 for the as-prepared He-charged zirconium films synthesized in pure He plasma. Nano-bubbles from 5 to 25 nm (marked by arrows) are clearly observed almost homogeneously spread over the thickness in this sample. Additional images obtained by SEM-EDS measurements have been included in Fig. 3s of the supporting information file for the Zr:He + Ar-DC sample after annealing. The EDS spectra couple to elemental mapping of Si (as substrate) and Zr from the film, confirm the strong flaking for this sample under thermal annealing.

## Discussion

In this work we used the MS deposition methodology in helium plasmas for the fabrication of solid–gas nanocomposite films of Co, Si and Zr. Helium incorporation reached high values in the range of 1 × 10^18^ to 3 × 10^18^ contents (in number of atoms per cm^2^ units). In terms of atomic percentages, He contents achieved a maximum of 42 at% value for sample 3 (Si:He-RF). The fabrication of solid–gas nanocomposite films is therefore clearly demonstrated. The helium content is measured by the IBA technique in a macroscopic area covering matrix and pores. However, from the previous results using EELS (Electron Energy Loss Spectroscopy) in a TEM microscope^30^, we know that helium is located inside the pores as also reported for Helium implanted samples^30^. Helium in the matrix if any is not relevant. In addition, the smaller pores stabilize the higher densities and pressures of trapped He (nanobubbles)^30^, therefore a direct correlation of porosity with the measured total He content is not straight forward. The microstructural characterization in SEM, and at higher magnifications in TEM, allows to identify contrast details which correspond to He bubbles as described in the previous He implantation works^1–5^. Alternatively, we describe the He bubbles in these films as He filled nanopores formed during the MS deposition procedure^30^. For the case of the cobalt matrix, we may first notice that pores are larger, and the amount of trapped Helium is smaller, as compared to values found for the silicon matrix. It is interesting to note that the microstructures of Co and Zr He-charged films are very different, even at similar He content (E49 and G2). This indicates that the different deposition conditions play a major role in the trapping and releasing processes of He in magneton sputter deposition.

Starting from this description of the solid–gas nanocomposite films we also present in this paper the study of the He release elucidating the evolution of the microstructure. For the different matrix elements, different nanostructures are formed after helium desorption. It is worth to emphasize the simultaneous recording of the helium and matrix-element IBA signals in a dedicated chamber for “in situ” thermal evolution in vacuum. Different behaviors are described for the different matrix elements. One of the main results obtained in this work is that blistering (flaking), only evidenced in DC sputtered films (Zr and Si), appears as a consequence of higher stress for the films fabricated in such conditions^25^. It is well known that the energy distribution functions of film forming species (depositing atoms and neutralized backscattered He ions) are different in RF and DC discharges. Therefore, for the silicon films the effect of the microstructure in the as-prepared sample **2** (Si:He-DC), incorporating both pores and blistering (Fig. 3), is likely affecting its lowest temperature when He release starts (515 K). For sample **3** (Si:He-RF), although with similar He/M ratio, the reduced blistering lead to stable films up to 625 K. For the case of sample **4** (Si:3He-RF), with the lowest He/M ratio, He release coupled to delamination starts in the range 673 to 723 K. For the case of a cobalt matrix, it is worth to mention the strong deformation of the matrix during He release under thermal annealing.

In conclusion, the here investigated thermal annealing procedure is leading to the fabrication of nano-porous and nano-structured films opening the scope for applications. In perspective the materials investigated in this work are expanding applications. An example are the recent reports of the use of porous amorphous Silicon^31^ and Germanium^32^ films as anodes for all-solid-state lithium batteries. The nanostructured films (fabricated by He assisted MS) can relieve deformation-induced stress and therefore enhance cycling performance.

## Methods

### Films fabrication by magnetron sputtering in He plasma

#### Co and Si films charged with helium

These films were prepared in a magnetron sputtering (MS) deposition chamber (residual vacuum in the range 1 × 10^–4^ Pa), operated with magnetron heads for 2 inches cathodes placed on top of the sample holder. For the preparation of the Co film, a magnetron from de AJA (USA) company was used with the adequate magnets configuration to work with magnetic targets. For the Si films we used a magnetron ION’X from the TFC (Germany) company. For operation power supplies from CesarRF-Dressler and Advance Energy-Pinnacle Plus were respectively used in RF and DC mode with constant power. The sample holder is at floating potential and was not cooled during the process. To estimate and monitor the substrate temperature during film growth, a thermocouple is placed in a lateral zone of the sample holder a few millimeters above the surface. The Si and Co targets were supplied from Neyco respectively with 99,999% and 99,95% purity. The distance from target to substrate was 8 cm for Cobalt and 10 cm for Silicon. As process gas we used He (and Ar for targets cleaning) supplied by Air Liquid with 99,999% purity. The 3He was supplied by Chemgas (≥ 99.9% purity). Table 1 summarizes the nomenclature of investigated samples along with their deposition parameters (gas pressures, power and time).

#### Zr films charged with helium

Films were deposited onto (100) oriented p-doped Si wafers by sputtering a 4-inch. Zr target (99.999% purity from Neyco). The substrates were pasted on a rotating substrate holder, and the distance between the substrate and the target was fixed at 12 cm. The system was kept under vacuum, reaching a base pressure of about 6 × 10^–6^ mb. The total pressure in the chamber was settled to 1 Pa using a gate valve before turning on the discharge. The Ar and He gas flow rates were adjusted using two Bronkhorst EL-FLOW mass flow controllers. The discharge current was regulated and set at 1 A using a DC power supply (Pinnacle plus from Advanced Energy). When the plasma is initiated in pure He, the resulting power was 300 W. In such working conditions, because of the discharge characteristics and of the very low sputtering yield, the deposition rate is very limited (about 2 nm/min as reported in Table 1). To study films of comparable thickness we decided to synthesize a second Zr sample, using a gas mixture containing 95% of He and 5% of Ar. As reported in reference for Al^11^, with such a gas ratio, the deposition rate is significantly increased, while He is still incorporated inside the film, even at higher percentages than in pure He plasma.

Co and Zr films were grown on 100 Si wafer substrates (0.5 mm thick) provided from Neyco. Si films were grown on the same Si substrates and on glassy carbon substrates (0.5 mm thick) also provided by Neyco.

### Films characterization by IBA analyses

For the as prepared samples the elemental analyses were done in two laboratories: (i) At the National Centre for Accelerators (CNA, Seville, Spain) for the helium charged Co and Si films. Proton elastic back scattering (p-EBS) was employed using a 2.0 MeV proton beam and a passivated implanted planar-silicon (PIPS) detector set at 165° for sensitivity to ^4^He and the matrix elements Si and Co. For the particular case of sample (Si:^3^He-RF) the p-EBS was carried out with the planar-silicon (PIPS) detector set at 159° for sensitivity to ^3^He. (ii) At the Pelletron accelerator of CEHMTI laboratory (Orléans, France) for the helium charged Zr films. Rutherford back scattering (RBS) and p-EBS were carried out using respectively: an α-beam at 2 MeV, 75° incidence angle and 166 ^o^ scattering angle for sensitivity to zirconium and a proton-beam of 2400 keV, 0° incidence angle and 178° scattering angle for sensitivity to ^4^He. Data analyses were performed by simulations with the SIMNRA code^33^.

### *“*In situ*” IBA analyses during annealing in vacuum*

Thermal annealing experiments were done for all samples included in Table 1 in the dedicated DIADDHEM chamber^34^ at the Pelletron accelerator of CEHMTI laboratory. p-EBS spectra were *in-situ* recorded each 1.5 min with a 3.4 MeV protons beam during continuous heating starting from room temperature with a 10 K/min ramp. Depending on the kinetic of He release final temperature, and eventual isothermal annealing, were selected.

### Films characterization by SEM and TEM analyses

The thickness and microstructure of the Co and Si films (before and after annealing) were examined by scanning electron microscopy (SEM) employing a HITACHI S-4800 SEM-FEG microscope operated at 1–2 kV. Thickness and morphology of the Zr films were determined by scanning electron microscopy (SEM) using a Zeiss Supra 40 FEG-SEM operating at 3 kV. In addition, Energy Dispersive X-ray Spectroscopy (EDS) at the accelerating voltage of 15 kV was carried out with a detector Bruker QUANTAX to draw a chemical cartography after annealing. Samples deposited on silicon or glassy carbon substrates were analyzed on top and cleaved for cross-sectional views. The nanostructure of the Co and Si nanocomposite films was investigated at the Laboratory of Nanoscopies and Spectroscopies (LANE-ICMS, Sevilla, Spain) by Transmission Electron Microscopy (TEM) using a Jeol 2100Plus TEM operated at 200 kV. For the Zr samples an ARM CFEG JEOL TEM working at 200 kV and equipped with 2 Cs aberration correctors was used. The cross-sectional TEM lamellas were prepared by mechanical polishing and dimple grinding of the coatings deposited on silicon or glassy carbon, followed by Ar^+^ ion milling to electron transparency. Representative porous areas were selected for imaging and analysis.

## Supplementary Information

Supplementary Information.

## Data Availability

The datasets used and/or analysed during the current study are available from the corresponding author on reasonable request.
