# Supplementary material for: Microstructure and composition evolution of He charged solid-gas nanocomposite films of different matrix elements during thermal annealing in vacuum
Source: Sci Rep. 2025 Jul 2;15:22935. doi: 10.1038/s41598-025-06889-8 (PMC12218580; doi:10.1038/s41598-025-06889-8)

SUPPORTING INFORMATION FILE

Microstructure and composition evolution of He charged solid-gas nanocomposite films of different matrix elements during thermal annealing in vacuum

Asunción Fernández*[1], M.Carmen Jiménez de Haro[1], Dirk Hufschmidt[1], Olga Montes[1], Thierry Sauvage[2], F.Javier Ferrer[3], Amaël Caillard[4], Pascal Brault[4], Anne-Lise Thomann[4].

[1] Institute of Materials Science of Seville(CSIC-Univ. Seville), Avda. Américo Vespucio 49, 41092-Sevilla, Spain.
[2] CEMHTI Laboratory, CNRS-UPR3079, 1D Avenue de la Recherche Scientific, 45071, Orléans, France.
[3] National Center of Accelerators, CNA (Univ. Seville, J. Andalucía, CSIC), Avda. Tomas Alva Edison 7, 41092, Seville, Spain.
[4] University of Orléans – CNRS, GREMI-UMR7344, 14 rue d'Issoudun, 45067, Orléans, France

- asuncion@icmse.csic.es

Fig. 1s: Representative X-ray diffractrograms for samples **1**. Co:He-RF (left) and **2**. Si:He-DC (right).
(a) The cobalt sample show the characteristic driffraction peaks for a crystalline film.
(b) For the silicon sample the lines show the position where the diffraction of the Si [111] planes should appear. The film appears amorphous.

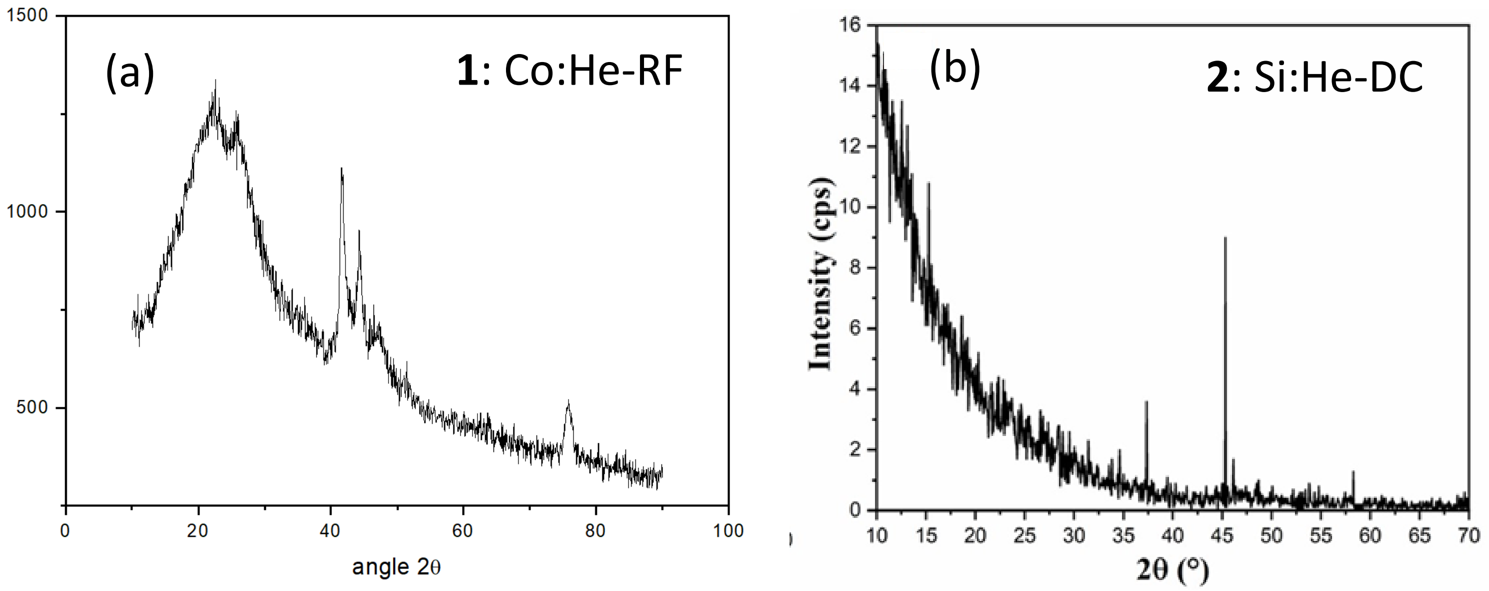

Fig. 2s: Cross section SEM images of representative areas of sample **2:** Si:He-DC after annealing. Blister formation is visualized. Blisters of different seizes were observed.

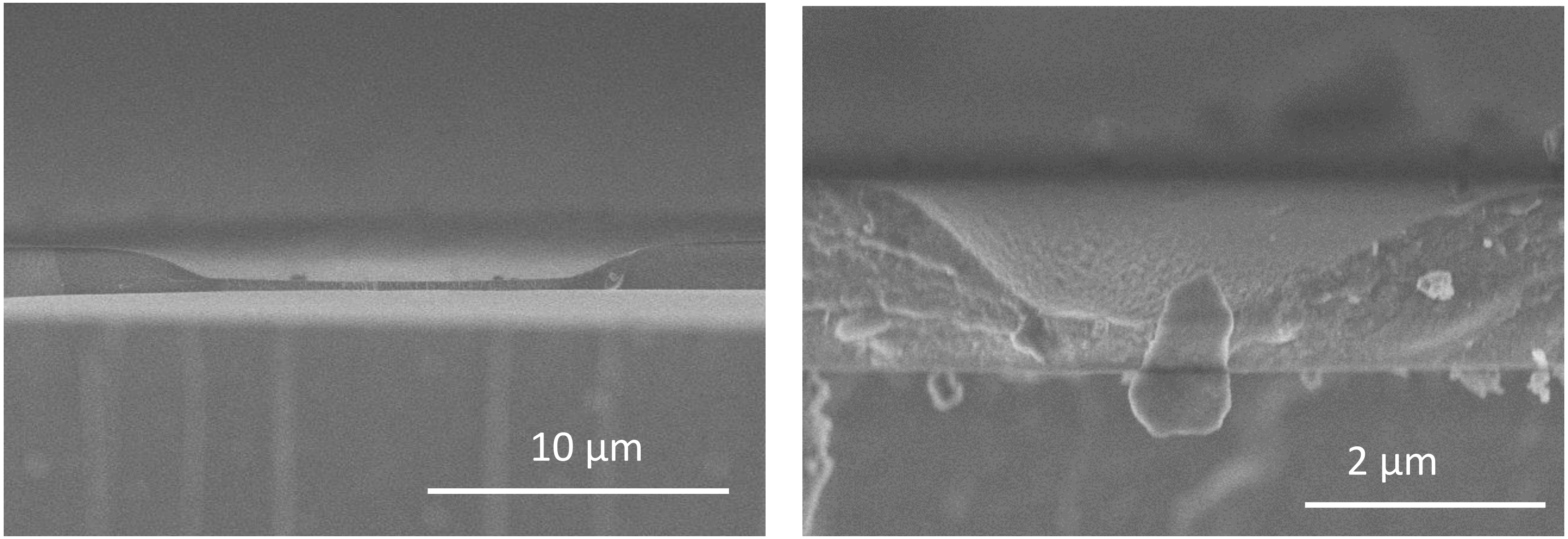

Fig. 3s: SEM-EDX analyses of sample **5.** Zr:He+Ar-DC after annealing: (a) Top-view SEM image. (b) Representative EDX spectra at positions 1 and 2. (c) Zirconium and (d) Silicon EDX maps for the square area selected in (B).

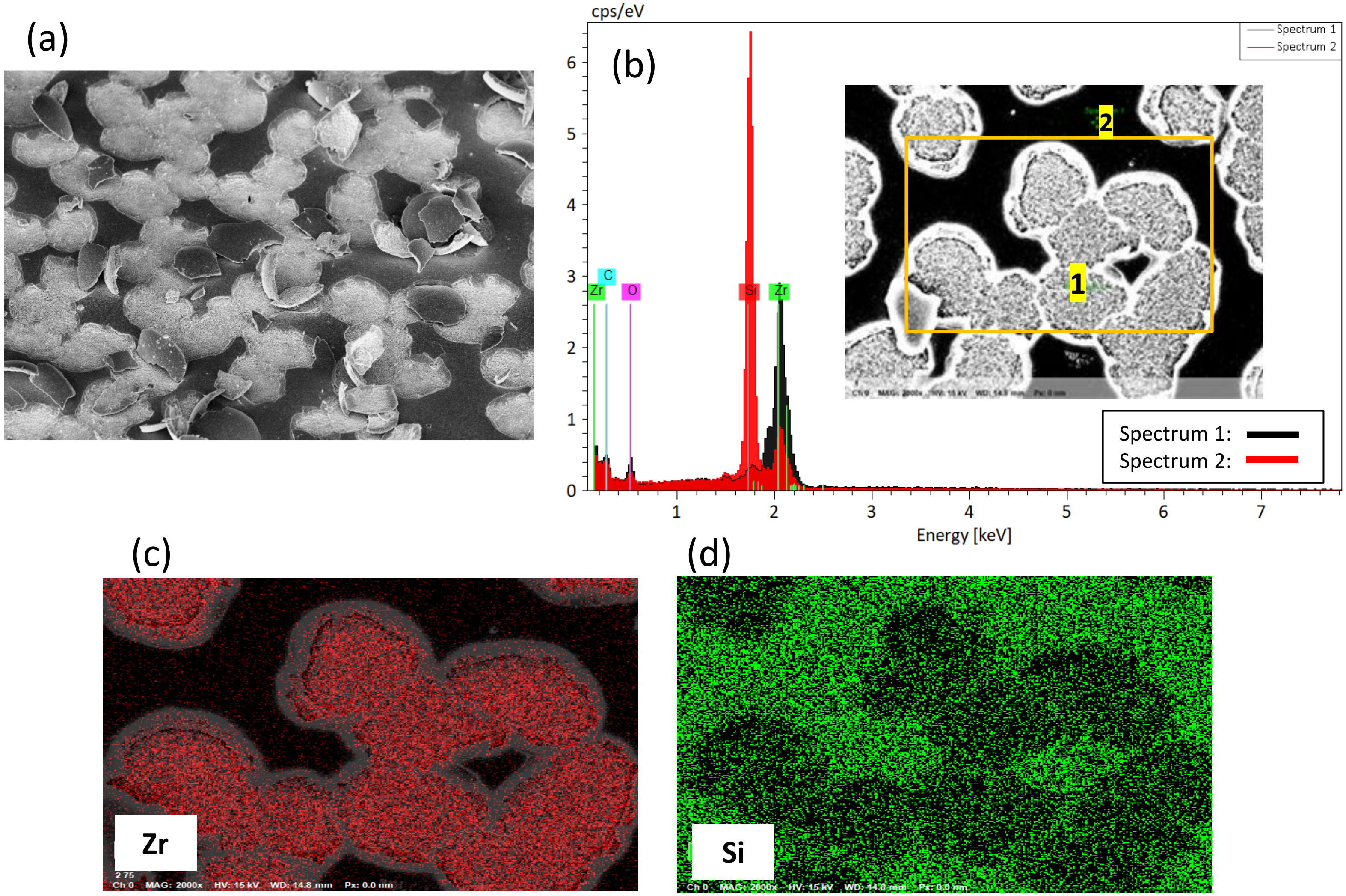

Supplement: Supplementary file 1 — Supplementary Information. [file 41598_2025_6889_MOESM1_ESM.pdf]
